# Integrating exposure to palliative care in an undergraduate medical curriculum: student perspectives and strategies

**DOI:** 10.5116/ijme.58cc.500c

**Published:** 2017-04-26

**Authors:** Adrian Y.S. Lee, Brodie Carlon, Rosemary Ramsay, Thiru Thirukkumaran

**Affiliations:** 1 School of Medicine, Rural Clinical School, University of Tasmania, Tasmania, Australia

**Keywords:** Integrating, palliative care, undergraduate medical curriculum, student perspectives, Australia

## Introduction

Palliative care (PC) is an integrated and holistic approach to the management of life-threatening or terminal illnesses through the prevention, assessment and treatment of distressing symptoms. It is a field in medicine that strives to encompass the whole patient and address their physical, psychosocial, emotional and spiritual care.  Because of the rise in the acknowledgement and presence of PC in clinical practice over the last 40 years, there is demand for more quality PC exposure and teaching in medical student education.^1^ PC education has also been criticised as being somewhat fragmented and internationally, there is little consensus of what exactly should be taught.^1^ In addition, concerns have been raised about students’ PC knowledge with one cohort of medical students scoring low on tested knowledge.^2^

Given the above concerns, we decided to establish medical students’ knowledge, understanding and perspectives on PC education at our medical school and in the literature to establish baseline views of PC education and curriculum. We established that low PC exposure is an area of urgent attention in student curriculums and strategies to address this have been proffered.

### Student Perspectives

At the Tasmanian School of Medicine (University of Tasmania, Australia), PC teaching is integrated throughout the undergraduate medical course with concepts of death and dying introduced early in Year 1, to nursing home and PC clinical attachments in Year 5. As part of a curriculum review process, we surveyed our undergraduate medical student cohort in 2015, using an internally-validated open survey.

We established that most students recognise and value the role of comfort care and alleviation of distressing symptoms as being integral to PC. They also remarked of the importance of biopsychosocial care, maintaining quality of life, communication and teamwork. When asked about concerns about their PC education so far, some concerns were raised about their overall low academic and clinical exposure, and correspondingly, some were concerned about their overall confidence in key skills in PC practice (for example, breaking bad news).

Previous studies are in agreement with our results, and show that medical students, in general, attribute high importance to palliative care in medicine, PC education, the psychological aspects of it, and learning about symptom control.^3^^,^^4^ However, there appears to be a mismatch between attitudes and the exposure of PC with relatively low clinical and academic exposure to PC noted in medical student curricula.^5^^,^^6^ Part of the reason may lie in the preference of medical school curricula for PC specialists to deliver academic teaching, rather than integrate their teaching with other specialties.^7^

Correspondingly, our survey of our medical students found a recurring theme of students requesting more and earlier clinical placements and exposure to PC. One student remarked: 

“I do not think that it is too early to implement more palliative education, as I can imagine it could be an extremely difficult situation to deal with, and the bigger skill base we have to deal with it, the less difficult it would be”.

Some also suggested further teaching in the discipline, with a few students pointing out that it would be ideal to see an experienced clinician demonstrate (with real patients) sensitive communication or PC principles.

### Evaluation and Strategies

It is clear from our survey and the literature that there is a general paucity of PC exposure in medical education. This is reflected in a number of recent initiatives to reform PC curricula and increase student exposure to the subject area.^8^ Attention to early exposure to PC would be vital for encouraging positive attitudes towards the subject area amongst students, and playing a role in their emotional and professional development as future empathetic practitioners.^9^^,^^10^ 

As a result, a determined effort by medical educators to incorporate PC elements into their clinical teaching should be made. A number of international medical schools have already introduced PC-oriented small-group discussions, workshops and reflective practice to promote learning and exposure to PC. Based on feedback questionnaires, these initiatives appear to have high student satisfaction scores and improve self-rated knowledge and skills in the area.^11^

To address clinical exposure, a possible strategy is to integrate inter-professional learning in PC clinical placements. This mode of learning combines the benefit of students from multiple health disciplines and promotes efficient collaborative practice, trust and reflective practice. At our medical school, we have recently introduced such an experiential learning initiative in PC and nursing home placements. Formal and informal feedback from our medical students has demonstrated this to be a favourable and enjoyable venture and something that they would continue in the future. We have plans to review learning outcomes of inter-professional learning in order to integrate assessment of collaborative skills and communication in future placements.

Similar to this problem of experience, a United Kingdom national study of medical school PC curriculum coordinators also revealed that a major problem for educators is establishing a suitable framework for adequate academic and clinical exposure. They reported that one of the biggest barriers is an already congested medical curriculum.^12^ Attention should therefore be focused on curriculum design at a course level in order to integrate elements of death and dying into academic and clinical teaching. Teachers may also adopt “incidental” PC teaching by demonstrating key PC skills in practice (e.g., eliciting the presence of distressing symptoms), and getting students to reflect on what they have observed.

A further strategy is the development of assessments (formative and summative) that are constructively aligned – this would seek to encourage students to develop essential “real-world” knowledge as they engage in their PC studies, and build their confidence in executing PC-related skills. Objective structured clinical examinations (OSCE) introduced in our medical curriculum have attempted to make this bridge and test students on “difficult” encounters in PC such as family meetings with conflict.

## Conclusions

The results of numerous studies and our survey of medical students demonstrate the overall favourable attitude that they hold towards PC in medicine. The biggest shortcoming appears to be the suboptimal academic and clinical exposure in PC education. Given that exposure is associated with increased positive attitudes and satisfaction with education,^9^ it remains clear that one of the key strategies to improve PC education and practice is through early and ongoing medical student exposure.

Our opinion with teaching PC is that medical educators should make a determined effort to incorporate PC elements into academic and clinical teaching, as well as assessment design. Granted, the vast knowledge and skills that is demanded from medical students during medical school makes this a major challenge. Therefore, ongoing curriculum reviews and seeking feedback from students is necessary in order to optimise PC exposure for students. 

### Acknowledgements

We would like to thank Professor M. Ashby and Associate Professor L. Shires from the University of Tasmania, as well as the staff of Palliative Care Service (North West) for their critical input into the questionnaire.

### Conflict of Interest

The authors declare that they have no conflict of interest.
